# When Doctors and Parents Don’t Agree: The story of Charlie Gard

**DOI:** 10.1007/s11673-017-9814-9

**Published:** 2017-11-06

**Authors:** Natasha Hammond-Browning

**Affiliations:** Southampton Law School, Southampton University, Highfield, Southampton, SO17 1BJ United Kingdom

**Keywords:** Withdrawal of medical treatment, Best interests of child, Parental wishes, Access to experimental treatment, Travel abroad for medical treatment, Article 2, 5, 6 and 8 ECHR

## Abstract

This discussion follows a series of high profile cases involving a terminally ill child, Charlie Gard. These cases are significant as they trace the complexities that arise when parents and medical teams do not agree as well as addressing the question of whether there is a right to access experimental treatment.

## Introduction

This discussion follows a series of high profile cases involving a terminally ill child, Charlie Gard. These cases are significant as they trace the complexities that arise when parents and medical teams do not agree as well as addressing the question of whether there is a right to access experimental treatment. At its heart, it is a story of human suffering, hope (and despair), and how a court can manage situations of unreconcilable differences of opinion regarding what is in the best interests of a child.

## Timeline

The legal case involving Charlie Gard, Great Ormond Street Hospital (GOSH), and Charlie’s parents Constance Yates and Chris Gard, was heard at all levels of the U.K. courts before progressing to the European Court of Human Rights and finally returning to the Family Division of the High Court. It was a lengthy process and before considering the key legal issues it is worth noting the timeline as it is complex, involves all levels of domestic courts, the European Court of Human Rights, the Pope, and the President of the United States—running in parallel with active social and mainstream media campaigns:

- 24 February: Application by GOSH to Family Division of High Court
- 3 March 2017: Mr Justice Francis starts to analyse the case at a hearing in the Family Division of the High Court in London
- 11 April: Mr Justice Francis grants declarations which allows doctors to stop providing life-support treatment as treatment is no longer in Charlie’s best interests
- 3 May: Charlie’s parents ask Court of Appeal judges to consider the case
- 23 May: Court of Appeal analyse and dismiss the case
- 8 June: Supreme Court dismisses the appeal
- 19 June: Supreme Court grant a further stay to the declarations for three weeks
- 20 June: Judges in the European Court of Human Rights consider written submission from lawyers representing Charlie’s parents
- 27 June: Judges in the European Court of Human Rights refuse to intervene
- 3 July: The Pope and U.S. President Donald Trump offer to intervene
- 7 July: GOSH applies to the High Court for an affirmation of the April declarations
- 24 July: Charlie’s parents end their legal fight to take him to the U.S. for treatment
- 26 July: Deadline set for Charlie’s parents and GOSH to agree how and when he will die
- 27 July: The 12pm deadline for Charlie’s parents and GOSH to agree how and when he will die passes
- 28 July: Charlie’s death is announced

## Factual Background

Charlie was born apparently healthy and at full term on August 4th, 2016. Over time it was observed that he was less able to lift his head or support himself than expected and was failing to gain weight. He was admitted to GOSH on October 11th, 2016 and subsequently diagnosed with a fatal and rare inherited mitochondrial disease called infantile onset encephalomyopathic mitochondrial DNA depletion syndrome, referred to generally as MDDS, caused by a mutation in the RRM2B gene.

Charlie’s parents accepted that his quality of life was poor and not worth sustaining but disagreed with the assessment of the team at GOSH that further treatment was futile and that palliative care should be pursued. Instead, they wished to take Charlie to the United States for experimental nucleoside therapy treatment. This therapy was in early stages of development and had yet to be tested on mice, let alone patients with the same strain of MDDS as Charlie.

## The First Hearing: *Great Ormond Street Hospital v Yates and others* [2017] EWHC 972 (Fam)

The medical experts at GOSH were firm in their rejection of the proposed therapy, arguing that it was not in Charlie’s interests and sought court intervention. On February 24th, 2017 GOSH made an application to the Family Division of the High Court seeking the following declarations:

1. That Charlie, by reason of his minority, lacks capacity to make decisions regarding his medical treatment;
2. That it is lawful and in Charlie’s best interests, for artificial ventilation to be withdrawn;
3. That it is lawful, and in Charlie’s best interests, for his treating clinicians to provide him with palliative care only; and
4. That it is lawful, and in Charlie’s best interests, not to undergo nucleoside therapy provided always that the measures and treatments adopted are the most compatible with maintaining Charlie’s dignity.

## The Law

Charlie clearly lacked capacity to consent or refuse medical treatment by reason of his age and his condition. In such circumstances it is usually the parents who make medical decisions, however, where there is intractable dispute between parents and medical professionals the final arbiter is the court; the overriding control is vested in the court exercising its independent and objective judgment in the child’s best interests. In making his decision in this instance, Francis J was guided by the “intellectual milestones” set by the Court of Appeal in *Wyatt v Portsmouth NHS Trust* [2000] 1 FLR 554. The Court of Appeal in *Wyatt* said:

In our judgment, the intellectual milestones for the judge in a case such as the present are, therefore, simple, although the ultimate decision will frequently be extremely difficult. The judge must decide what is in the child’s best interests. In making that decision, the welfare of the child is paramount, and the judge must look at the question from the assumed point of view of the child. There is a strong presumption in favour of a course of action which will prolong life, but that presumption is not irrebuttable. The term “best interests” encompasses medical, emotional, and all other welfare issues. ([38])

## Medical Evidence from GOSH

Medical experts from GOSH explained that due to MDDS Charlie had suffered dysfunction of several organ systems; his brain, muscles, and ability to breathe were all severely affected. In addition, he had congenital deafness and a severe epilepsy disorder. His heart, liver, and kidneys were also affected although not severely. Charlie had severe progressive muscle weakness and could not move his arms or legs or breathe unaided. He was persistently encephalopathic so, whilst not brain dead, there were no usual signs of normal brain activities such as responsiveness, crying, or interaction and he suffered from seizures. The medical experts were in agreement that there was no chance of recovery and that Charlie did not derive any benefit from continued life. As noted by Francis J, Charlie was in a parlous state and MDDS is a progressive and life-limiting condition. There was no evidence of Charlie responding to his parents, it was impossible to tell if Charlie was awake or asleep, or to know if he was suffering pain, pleasure, or comfort.

## The Proposed Treatment: Nucleoside Therapy

Charlie’s parents did what many would do, they searched for a treatment that would prolong life or even slightly improve Charlie’s condition. They discovered nucleoside therapy, which had been used on patients with a different and less severe mitochondrial condition known as TK2 mutation. There was some evidence that patients with the TK2 mutation had benefited from nucleoside therapy.

The parents were in contact with Dr I, a professor of Neurology at a medical center in the United States, regarding possible treatment of Charlie. Initial discussions confirmed that there was a “theoretical possibility” of the nucleoside therapy being of some benefit to Charlie but Dr I made it clear that a baseline MRI scan was necessary as severe brain involvement was a contraindication to the therapy being trialled (*Great Ormond Street Hospital v Yates and others* [2017] EWHC 972 (Fam)[77]). In January 2017, following a further MRI that appeared to show no structural damage, GOSH drafted an Ethics Committee application so that nucleoside therapy could be considered in England. Charlie was also placed on a list for a tracheostomy to be performed on January 16th, 2017.

It was at this time that disagreement between GOSH and the parents emerged. Charlie suffered an episode of seizure activity that started on January 9th or 10th and continued intermittently until January 27th. The Ethics Committee meeting set for January 13th was postponed due to the increased seizure frequency and likely severe epileptic encephalopathy. On January 13th, Charlie’s consultant neurologist, Dr K, and his ICU consultant met with his parents to inform them that Charlie was suffering with severe epileptic encephalopathy and that all teams were in agreement that the nucleoside therapy would be futile and only serve to prolong Charlie’s suffering. It was clear to the medical team that it was no longer in Charlie’s best interests to be subjected to nucleoside therapy, but Charlie’s parents did not agree.

It is worth noting here that GOSH has asserted that funding was not an issue and if nucleoside therapy had been of benefit to Charlie then it would have pursued that treatment. This is contrary to the view that many others formed in the context of the parents’ passionate and successful plea to the public to provide funding for treatment in the United States, with over £1.3million raised via a gofundme page (GoFundMe 2017).

Crucially, it was presented to the court that even if nucleoside therapy was able to cross the blood/brain barrier (which was essential in this situation), it was not possible to reverse the process for neurones already lost. It was predicted that death was at most six to nine months away. One medical expert, Professor A, asserted that there was a difference in philosophy of treatment in the United States and in the United Kingdom. She suggested that in the United States any medical treatment will be attempted so long as funding is available, whilst her approach was one that centred on the best interests of the patient (*Great Ormond Street Hospital v Yates and others* [2017] EWHC 972 (Fam) [90]). The U.S. medical expert Dr I made it clear that, despite the lack of scientific evidence of potential improvement in Charlie’s specific circumstances, he would treat him ([99] and [106]). Of significance to the court, Dr I described the probability of benefit to Charlie’s brain as low but not zero and agreed that there could be no reversal of the damage to Charlie’s brain.

## Position of The Parents

The parents did not accept that Charlie was as bad as reported by the medics, and did not want the declarations to be granted. They wanted to give Charlie a chance to improve.

## Position of Charlie Through His Guardian

Charlie’s Guardian, Ms Butler-Cole, concluded that “it is not in Charlie’s best interests to travel to America to receive nucleoside therapy. This is not pioneering or life-saving treatment, but purely a experimental process with no real prospect of improving Charlie’s condition or quality of life” (*Great Ormond Street Hospital v Yates and others* [2017] EWHC 972 (Fam) [117]). The presented evidence prompted Francis J “to ask the question as to whether it would be worth giving it a try on the basis that, without experimentation, medicine cannot advance” ([119]). Significantly however, as noted by the judge, the legal test that he had to apply “ … is what is in Charlie’s best interests not what is in the best interests of medical experimentation” ([121]) thus rendering his question obsolete.

## Decision

Francis J granted the declarations sought by GOSH; it was not in Charlie’s best interests to have nucleoside therapy and therefore it was lawful to withdraw artificial ventilation and provide palliative care only.

The withdrawal of treatment was delayed until the parents had made a decision regarding the appeals process.

## The First Appeal, Challenging Best Interests: *Yates and Gard v Great Ormond Street Hospital* [2017] EWCA Civ 410

Charlie’s parents appealed seeking a positive declaration that they could arrange for Charlie to be transferred to a clinic in the United States to receive nucleoside therapy. They were represented by a new legal team which presented five grounds of appeal, three of which the Court of Appeal felt were sufficient to hear but all were ultimately unsuccessful.^1^

Grounds 1 and 2 before the court centred on procedural issues and focussed on the jurisdiction of the court and assertions that, as a matter of law, a different approach should be taken to cases where a choice is to be made between two viable treatment options. Ground 4 of the Appeal was the claim that no, or insufficient, regard was had to the rights of Charlie and his parents under Articles 2, 5, and 8 of the European Convention on Human Rights; this last ground was granted permission to appeal on the basis that the arguments supported the other two grounds for appeal.

The permitted grounds of appeal raised new legal argument as well as a new point of law. First, it was claimed that “The judge had erred in making an order that prevented C from receiving medical treatment by expert physicians in a reputable hospital overseas in circumstances where there was no risk of that treatment causing significant harm to C” (Ground 1). Secondly, that

The judge had no jurisdiction to grant an order on the application of one clinical team preventing a second clinical team from carrying out a treatment that the latter had offered in the reasonable exercise of its professional judgment … The declaration made by the judge has de facto injunctive effect in that it prevents C’s parents from removing from GOSH to undergo treatment in the USA. (Ground 2) (*Yates and Gard v Great Ormond Street Hospital* [2017] EWCA Civ 410[54]).

It was argued that there should be two categories of cases in instances of dispute about medical treatment of a child; Category 1 involved situations where no alternative therapeutic option was presented and should be dealt with under the best interests test, and Category 2 where a viable alternative was put forward by the parents, in which the court should be required to choose between the proposed treatments. It was argued that parental choice should only be overridden if the proposed option was likely to cause the child to suffer significant harm, even if the proposed course of treatment is not in the child’s best interests ([110]). *Re King* [2014] EWHC 2964 (Fam) was presented as the only authority for this distinction.

The Court of Appeal rejected this distinction and upheld the primacy of the best interests of the child test. The Court concluded that *Re King* provided no basis for any test based on significant harm to be applied to cases relating to the medical treatment of children and that “the sole principle is that the best interests of the child must prevail and that must apply even to cases where parents, for the best of motives, hold onto some alternative view” ([112]). As noted by MacFarlane LJ, the argument was also undermined by the fact that “There is no viable, alternative treatment for poor C,” and that if invited to do so, the judge would have formed a conclusion that Charlie was currently suffering significant harm and “that to move C to America and expose him to treatment over there would be likely to expose him to continued pain, suffering and distress” ([113] and [114]).

With regards to Ground 2 it was noted that GOSH made the application to the court in the conventional terms. The issue of alternative therapy was one that was raised by the parents as the proceedings progressed, at which point the choice had to be made by the judge rather than the hospital ([116]).

## The Second Appeal—A Further Challenge to Best Interests Test: *In the Matter of Charlie Gard,* Supreme Court, June 8th 2017

There was a further attempt to challenge the conclusions on the basis that the best interests test was not correct and that the hospital could only interfere in the decision taken by the parents if the child is likely to suffer significant harm. It was also argued that decisions taken by parents who agree with one another are non-justiciable as parents alone are the judges of their child’s best interests; any other approach would be an unjustifiable interference with their status as parents and their rights under article 8 European Convention on Human Rights. These arguments were rejected and the primacy of the best interests test re-asserted.

## On the Way to The European Court: *Judgment of the U.K. Supreme Court in the Case of Charlie Gard*, June 19th, 2017

The Supreme Court was involved in the legal proceedings once more due to the further appeal, this time to the European Court of Human Rights. The U.K. Government asked the Supreme Court to give directions that it considered appropriate with regard to the interim order under Rule 39 of the European Court of Human Rights. The interim order required the provision of artificial ventilation nutrition and hydration (AVNH) to Charlie until the European Court had determined the application (*Judgment of the U.K. Supreme Court in the Case of Charlie Gard*, June 19th, 2017 [5]).

The Supreme Court noted the difficulty that this further stay of declarations placed upon the court, the hospital, and overarchingly upon Charlie himself ([7]). Reference was made to the numerous successive stays granted since April 11th, all of which, evidence suggested, were not in the best interests of Charlie. As noted by the Supreme Court,

The hospital finds itself in an acutely difficult ethical dilemma: although the stays have made it lawful to continue to provide him with AVNH, it considers it professionally wrong for it to have continued for over two months to act otherwise than in his best interests (*Judgment of the U.K. Supreme Court in the Case of Charlie Gard*, June 19th, 2017 [15]).

These concerns were echoed by Charlie’s guardian who submitted that it was time for the court to decline any further stays. But given the history of previous stays and the appeal to the European Court of Human Rights, with hesitation, the Supreme Court granted a further stay of three weeks but urged the European Court to address the application within this time frame ([20]).

## And Finally: *Gard and Others v U.K.* ECHR app no 39793/17

The European Court heard (and rejected) arguments on behalf of Charlie and his parents. Charlie’s parents argued that, in blocking access to life-sustaining treatment, GOSH had deprived Charlie of his liberty in contravention of Articles 2 and 5 European Convention on Human Rights. Charlie’s parents further complained that under Article 6 the Court of Appeal conclusion that their intended parental decisions would cause Charlie “significant harm” was made without hearing witness evidence. And finally, they claimed under Article 8 to have suffered disproportionate interference in their parental rights because the High Court decision that had been taken on the basis of the best interests of a child, failed to ask whether there was a likelihood that the child “is suffering, or likely to suffer, significant harm” (*Gard and Others v U.K.* ECHR app no 39793/17 [55] and ([56]).

The foundational principle was once again seen to be the best interests of the child with the European Court of Human Rights concluding that the decisive issue was

… whether the fair balance that must exist between the competing interests at stake—those of the child, of the two parents, and of public order—has been struck, within the margin of appreciation afforded to States in such matters, taking into account, however, that the best interests of the child must be of primary consideration. (*Gard and Others v U.K.* ECHR app no 39793/17 [107])

The European Court of Human Rights recognized that the domestic framework has a wide margin of appreciation in cases that raise sensitive moral and ethical issues. Equally, there was a U.K. legal framework that was Convention compliant and governed access to experimental medication as well as withdrawal of life-sustaining treatment. The domestic court decisions had been meticulous, thorough, and reviewed at three levels of jurisdiction. The European Court gave significant weight to the fact that the U.K. court had had direct contact with all involved as well as access to significant technical expertise and concluded that it was not the role of the European Court to substitute itself for domestic courts.

## Trial by Judiciary or Trial by Public Opinion?

This series of decisions is a clear application and endorsement of well established legal principles to determine if medical treatment is in a child’s best interests, at each level the court took into account medical advice, parental wishes, the child’s current and future position, and the possibility of experimental medical treatment. In that regard, the decisions are legally unremarkable. What makes them noteworthy (and potentially concerning) is the parallel process of “trial by public opinion” fed by social-media campaigns and outside commentaries that culminated in the extraordinary intervention by both the President of the United States and the Pope. The courts on the one hand were carefully considering the medical evidence and the harsh realities of Charlie’s situation and weighing this against the hopes of parents confronted with the tragedy of a dying child. The public (and the Pope and the President) did not, however, have the luxury of meticulous evidence, they focused instead on the “rights of the parents” and challenged the role of the medical profession and judicial system in decision-making in this context. The great tragedy of a terminally ill child translated into a dialogue about parental rights and this was fuelled by the fundraising efforts of Charlie’s parents which was bolstered by media coverage (GoFundMe 2017).

Media reports described nucleoside therapy as “innovative,” “radical,” and “pioneering” (Smith-Squire and Roberts 2017; Al-Othman 2017; Telegraph Reporters 2017). These value-laden terms pointed to hope and cure but Charlie’s interests would have been better served with the more accurate description of “experimental.”^2^ The extensive testing, research ethics, and protocols that are in place for new treatments, medicines, and devices have been designed to ensure that treatments are safe and effective when they reach patients. Accusations of the law preventing innovative medical treatment are not justified (Richards 2016), and terming experimental treatments as “innovative” suggests that it is new but tried and tested; nucleoside therapy had not even been tested on mice. Charlie’s case has been aired extensively in the media across the world over the last few months; the media spin put on Charlie’s case did not help his medical situation, and it has been argued by at least one commentator that the inaccurate reporting in the United States on the situation “reinforced the parental refusal to accept this tragic situation” (Phillips 2017)*.*

In contrast to the media reports that supported the parent’s wishes to take Charlie abroad for experimental treatment, GOSH and Charlie’s treating doctors, alongside the legal system were presented as blocking life-saving treatment, removing the right of parents to make decisions regarding their children, and of not having Charlie’s interests as their paramount concern (Smith-Squire 2017). However, as explained, the courts consistently referred back to Charlie’s best interests and it was his parents and their supporters who argued that this was not the correct test to apply.

This series of cases raised many questions, and it became apparent that two potential imperatives drive access to treatment, the first being the best interests and the other, the “experimental”/“financial.” The willingness of the U.S. doctor to provide this experimental therapy raises ethical questions of providing a treatment purely based on the availability of funding. At some point Charlie’s parents were allowed to believe that if they could simply raise the money to travel to the United States then their son would have hope, whereas the willingness to provide an untested therapy on the sole basis of raising the required money should have raised warning flags and prompted further questions around anticipated outcomes. There was an implication that GOSH was refusing treatment based upon financial constraints. This was in direct contrast to assertions by GOSH that funding of treatment was never an issue, had taken steps towards seeking ethics committee approval in order to provide nucleoside therapy, and that all medical experts involved agreed that the therapy was futile.

Unfortunately, the legal saga did not end with the European Court of Human Rights decision; in early July the President of the United States, Donald Trump, and the Pope intervened by offering treatment to Charlie. On July 7th, GOSH returned to the High Court for affirmation of the declarations made on April 11th and requested that orders were made due to the interpretation by the White House that declarations would permit transfer of Charlie to another hospital (*GOSH v Yates and others* [2017] EWHC 1909 (Fam) [2]). It was asserted by the parent’s solicitors that new evidence meant that there was a duty upon GOSH to refer the matter back to court. This new evidence included that there was a hospital in Rome willing to accept the transfer of Charlie, as was the U.S. centre, and on the basis that Dr Hirano (previously referred to as Dr I) had new laboratory findings that indicated a higher possibility of benefit to Charlie as well as likelihood that the therapy would cross the blood brain barrier ([8]). Dr Hirano visited Charlie in hospital on July 18th. Charlie’s parents gave up the legal fight on July 24th after Dr Hirano examined a more recent MRI scan and concluded that Charlie had no chance of success from the nucleoside therapy. And then the tide of public opinion turned, and Dr Hirano has since been strongly criticized by the press, social media, and notably by GOSH (GOSH 2017)*.* It is not possible to know if agreement between the parents and GOSH would have been reached sooner if Dr Hirano had visited earlier, but his failure to engage fully with all available medical evidence before advising both the parents and the court was inexcusable (Jayaram 2017).

The legal proceedings and media circus did not, however, end when Charlie’s parents withdrew their opposition. There was a failure to come to an agreement about the timing and place in which Charlie’s withdrawal of treatment would occur. Charlie’s parents wanted to take him home for his last few days, but due to the invasive ventilation that Charlie was being treated with, GOSH was not in a position to enable this, not least because it was said that the equipment would not fit through the front door of their house. The court was again called on to intervene and Francis J was forced to give a deadline of 12pm on Thursday July 27th in order for GOSH and Charlie’s parents to come to an agreement, otherwise Charlie would be transferred to a hospice. The deadline passed without agreement. Charlie was transferred to a hospice, and his parents announced his death on July 28th.

This series of cases serve as a strong cautionary tale, sometimes those closest to a sick person are not in the best position to make decisions, tragedy and grief, combined with offers of hope can cloud judgement. It was clear, and this was emphasized by Francis J, Charlie’s parents were clearly dedicated to him, they were living every parent’s nightmare and were driven by a desperate hope but potentially failed to see what would be in his best interests. In complex situations such as this, it is vital that the Court acts as an arbiter to ensure that the child’s best interests are applied as the paramount concern. As demonstrated throughout the Gard cases, the courts will, at all times, pay respect to the treating team and the parents but above all, the patient at the centre of the dispute (here, it was Charlie) comes first. Sadly, this cannot be said of the poorly informed international public narrative that surrounded the cases, which is why we must look to the legal system and not social media to answer the difficult question of what is in a child’s best interest.

## Footnotes

1 The two grounds which were refused permission to appeal are Ground 3, the assertion that the judge fell into error in conducting the “best interests” evaluation, and Ground 5, that the parents and the legal team were placed at an unfair disadvantage as a result of late disclosure of documents. These were unarguable (*Yates and Gard v Great Ormond Street Hospital* [2017] EWCA Civ 410, [36]).

2 It is recognized that many media sources did refer to the proposed treatment as experimental although few referred to the fact that this treatment had not even been tested on mice. For example, the Daily Mail provided an extensive review of the court proceedings, but omitted the lack of research that the proposed treatment had gone through (Moore and Greenhill 2017).

## References

[CR1] Al-Othman, H. 2017. The High Court has ruled that this baby should be allowed to die. BuzzFeedNews, April 11. https://www.buzzfeed.com/hannahalothman/the-high-court-has-ruled-that-this-baby-should-be-allowed?utm_term=.uq28GA2vx#.iaW5QzqXD. Accessed April 14, 2017.

[CR2] GoFundMe. 2017. Please help to save Charlie’s life. https://www.gofundme.com/please-help-to-save-charlies-life. Accessed April 30, 2017.

[CR3] Great Ormond Street Hospital (GOSH). *GOSH’s Position Statement Hearing 24 July 2017*. http://www.gosh.nhs.uk/news/latest-press-releases/gosh-position-statement-issued-high-court-24-july-2017. Accessed July 24, 2017.

[CR4] Jayaram, R. 2017. Would Charlie Gard really have survived if he’s been treated sooner? Here’s the sad truth. *The Independent*, July 26. http://www.independent.co.uk/voices/charlie-gard-gosh-hirano-would-he-have-survived-a7861531.html. Accessed July 26, 2017.

[CR5] Moore, C., and S. Greenhill. 2017. Baby Charlie’s parents sob as court-appointed guardian tells judge that he should be allowed to die despite £1.2million raised to send him to US for treatment. *Daily Mail*, April 7. http://www.dailymail.co.uk/news/article-4389784/High-Court-judge-rule-fate-baby-Charlie-today.html. Accessed April 07, 2017.

[CR6] Phillips, M. 2017. A cruel and ignorant campaign. *MelaniePhillips.com*, July 26. http://www.melaniephillips.com/cruel-ignorant-campaign/. Accessed July 26, 2017.

[CR7] Richards, B. 2016. Medical innovation laws: An unnecessary innovation. *Australian Health Review* 40(3): 282–285. 10.1071/AH15081 26363768

[CR8] Smith-Squire, A. 2017. Charlie Gard’s parents condemn Great Ormond Street’s “inhuman” treatment of their son and say that they have been stripped of their parental rights as they release new picture at his bedside. *Daily Mail Online*, May 29. http://www.dailymail.co.uk/news/article-4553188/Charlie-Gard-s-parents-condemn-hospital-s-treatment-son.html. Accessed May 29, 2017.

[CR9] Smith-Squire, A., and S. Roberts. 2017. “If we were British our son would be dead” American couple whose son survived thanks to the therapy Charlie Gard’s parents are fighting for slam controversial court ruling. *The Sun*, April 12. https://www.thesun.co.uk/living/3318065/arthur-olga-estopinan-arturito-charlie-gard-court-case/. Accessed April 12, 2017.

[CR10] Telegraph Reporters. 2017. Charlie Gard’s mother asks judge to give critically ill baby “one shot at life.” *The Telegraph*, April 7. http://www.telegraph.co.uk/news/2017/04/07/charlie-gards-parents-set-discover-have-won-high-court-fight/. Accessed April 07, 2017.

